# Gambling among indebted individuals: an analysis of bank transaction data

**DOI:** 10.1093/eurpub/ckad117

**Published:** 2023-07-13

**Authors:** Virve K Marionneau, Aino E Lahtinen, Janne T Nikkinen

**Affiliations:** University of Helsinki, Centre for Research on Addiction, Control, and Governance (CEACG), Finland; University of Helsinki, Centre for Research on Addiction, Control, and Governance (CEACG), Finland; University of Helsinki, Centre for Research on Addiction, Control, and Governance (CEACG), Finland

## Abstract

**Background:**

Gambling is connected to important financial harms, including debt. Most existing research has investigated the relationship between gambling and debt using self-reported data. Only a few studies have used objective data. The current study focuses on the gambling of indebted individuals. It investigates the amounts and types of gambling consumed by indebted individuals, and the amounts of unsecured debt among heavy gamblers.

**Methods:**

We use past-year banking data of Finnish individuals (*N* = 23 231) collected between 2018 and 2021 among applicants to a debt consolidation service. The transactions consist of deposits to, and winnings paid by gambling operators, distinguished by type of gambling (sports betting, casino, lottery) as well as active loans divided into secured and unsecured loans.

**Results:**

Gambling is widespread among indebted individuals in Finland. In terms of gambling types, casino-type gambling is the most popular among indebted individuals. Gambling spending is highly concentrated. Nearly half (49.5%) of all gambling deposits are concentrated among the highest spending 5% of indebted individuals. Individuals with unsecured loans have higher median losses than those without unsecured loans.

**Conclusions:**

The results suggest that gambling and indebtedness are strongly linked. The connection is stronger for individuals with unsecured debt. This has implications for prevention and treatment. Easy access to unsecured credit is likely to worsen gambling harms. Debt counselling services routinely encounter gambling-related harms and need to be equipped to manage these issues.

## Introduction

Financial difficulties, and particularly debt, are among the most pressing negative consequences of gambling for individuals, families, and communities.^1^^,^^2^ Gambling-related debt refers to situations in which individuals borrow money to finance continued gambling, or to meet other financial commitments created by gambling losses. Financial debt can include various forms, including unpaid bills, arrears, overdrawn bank accounts, and loans.^3^^,^^4^

Previous literature has connected gambling to high use of instant loans and consumer credit.^5^ Borrowing money to gamble features as an item in assessment tools to measure the prevalence of problem or pathological gambling (e.g. South Oaks Gambling Screen (SOGS), Canadian Problem Gambling Inded (CPGI)). Research has shown that excessive gambling and debt are strongly connected.^4^^,^^5^^,^^6^ Those with clinical problem gambling often have high levels of debt^3^^,^^7^ and the severity of gambling problems correlates with the amounts of debt.^5^^,^^8^ Debt can be secured or unsecured. Secured loans are guaranteed by collateral and assets while unsecured loans, also called instant loans, do not require a guarantee but also charge higher interest rates. Survey evidence from the UK shows that those with problem gambling (Problem Gambling Severity Index (PGSI) 8+) finance their gambling using credit cards (38% versus 8% for those without gambling issues), overdraft (28% versus 1% for others), instant loans (20% versus 0% for others), and other loans (18% versus 0% for others). Debt is also the most important reason for help-seeking among gamblers^9^ and an important contributor to gambling-related suicidality.^10^

Most research exploring the connection between gambling and debt has used self-reported data^7^ that may be distorted due to self-reporting bias.^11^ One previous UK study used a large financial dataset (*N* = 6 500 000) to estimate the relationship between gambling and general financial harms.^12^ Based on these banking data, the study found that gambling was connected to foreclosures as well as higher credit card use. A 10% increase in gambling consumption was connected to a 11.2% increase in credit card usage, as well as a 51.5% increase in high-cost payday loans.^12^ The study was only able to analyse direct gambling payments and not payments made using third-party payment services or in cash (also Ref.^13^) Another UK study^14^ analysed clients of a consumer credit counselling service StepChange (*N* = 206 241 across 2019–21). Two percent of clients were flagged for gambling (*N* = 1559). These individuals had a considerably higher median of unsecured debt (£10 235) than those who were not flagged for gambling (£6033).

The current study analyses gambling spending of indebted individuals who have contacted a Finnish credit counselling and debt consolidation service (*N* = 23 231, data collected between 2018 and 2021). The analysis focuses on the amounts and types of gambling consumed by indebted individuals and the amounts of secured and unsecured debt among gamblers. The results are discussed in terms of their implications for the prevention and treatment of gambling-related financial harms.

## Methods

### Setting

The data for this study were provided by a private Finnish credit counselling and consolidation service Solidate (a non-listed company). Individuals with debt repayment issues can contact the service to request for assistance and a consolidation loan to repay unmanageable debt. Upon registration, individuals provide Solidate with access to their bank account transactions from the previous year (12 months) as well as information of accumulated credit. Solidate uses these data to determine whether the client is eligible for the consolidation scheme. For instance, heavy past year gambling (as determined by the company) or previous loan consolidation processes are flagged and result in a failed process.

Finland has comparatively high overall gambling participation and consumption. According to the latest population study from 2019, 78.2% of the population had gambled in the past year. However, most only play lotteries.^15^ Finland has a national monopoly on gambling, operated by Veikkaus. Notwithstanding the monopoly system, a significant part of online gambling is directed towards unlicenced offshore websites. The market share of the monopoly in the online channel was an estimated 59% in 2021.^16^

Unsecured instant loans are easily accessible in the Finnish context, but the instant loan industry has been regulated with interest rate caps. The cap is currently 20%, although it was temporarily reduced to 10% during the COVID-19 crisis. The conditions provided by debt consolidation services can therefore help individuals manage debt with unmanageable interest payments. Data from Finnish help and support services show that gambling with instant loans is the gambling-related harm most often mentioned by help-seekers.^17^

### Data source, participants and possible bias

The data consist of client data collected by Solidate as part of their debt refinancing operations. Solidate collects data on the socio-demographics, past year bank transactions and loans of their prospective customers. The data were collected between May 2018 and October 2021. The data consist of a total 23 231 individuals who have created a client profile with Solidate. The bank transaction data consist of gambling transactions of each individual in the 12 months preceding their application. Only gambling-related bank transaction data were transferred by Solidate for this research, due to raised data privacy issues during the data transfer negotiations.

Gambling is one of the main reasons why debt consolidation applications submitted to Solidate are dismissed. In total, 4006 debt refinancing applications in the data were rejected due to gambling based on an assessment made by Solidate. These individuals are included in the current data. Solidate also informs customers on their website that heavy gambling in the past 6 months will result in denial. This likely causes bias in the data, as individuals with heavy gambling might be less likely to apply for the service, and therefore might be less likely to be recorded in the data. The results may therefore be skewed towards individuals with less gambling than could be expected among indebted individuals.

### Variables

The data consist of variables collected in the operation of the debt consolidation service. Customers had not been informed of possible research-related use of their data. For reasons related to privacy protection and research ethics, only variables relevant to gambling consumption could be transferred for research use. For each individual in the data, we therefore obtained information on: (i) year of birth; (ii) past year gambling-related bank transactions consisting of deposits to gambling operators by type (sports betting, casino, lottery) and winnings paid; and (iii) active loans divided into secured loans (backed by collateral) and unsecured loans (e.g. instant loans). The full dataset includes 3 049 125 lines of gambling-related bank transactions and 34 101 lines of loan-related information.

The bank transaction data are classified under transaction codes by Solidate. The transaction codes are based on the categorization created by a fintech company utilized by Solidate to classify data. Outgoing payments (deposits) to gambling companies are electronically identified as gambling (86) and, when possible, further divided under lottery (4402), casino (4404), and sports betting (4406). When gambling-related payments were made using an online payment service, these were classified under other online payments (4408). Payment services are third-party intermediaries. When payment services are used, payments do not necessarily include a gambling company as a transaction partner or payment recipient. Solidate identified recurrent payments using payment services that are typically used by online gambling providers (Trustly, Pluspay, and Euteller) as gambling. When payment services were used, the type of gambling product could not be determined.

Winnings were similarly classified under gambling (236), and when possible, subdivided into lottery (4401), casino (4403), and sports betting (4405). When winnings were paid using an online payment service, these were classified under other personal transfers related to gambling (711) and lacked information on product type. These data are likely not to be complete, as some online payments have not been connected to gambling. This applies to both deposits and winnings. The scale of this limitation is not known because we did not access transactions that were not categorized as gambling. It is nevertheless likely that the limitation is not major, as it is in the interest of the loan consolidation company to identify gambling transactions with the best possible accuracy. Furthermore, land-based gambling is often not visible in bank transactions, if the individual has used cash or purchased gambling products in, for example, supermarkets. This skews the sample towards online forms of gambling.

### Statistical methods and measurements

Although Solidate collects different socio-demographic background information on their customers, only their year of birth was transferred for this research. This limited the possibilities for any analysis based on individual socio-demographic characteristics. The analysis therefore uses descriptive statistics on gambling consumption in the sample.

The data were initially transferred as an SQL dump file. We used SQLite to transfer the data into a database file. All analyses were then conducted in R using the dplyr and dbplyr packages. We first cleaned the data by removing outlying extreme values that were clear errors (over 1 billion euros). Besides clear errors, we did not remove extreme values, as large deviations are naturally present in gambling. Only in the case of unsecured loans, we set a limit of 500 000 euros for included single unsecured loans, removing 11 values. This decision was made because larger loans were deemed unlikely. To limit the effect of extreme values on results, the analysis focuses on median values.

The variables used in the data analysis were based on categories in the dataset. Gambling transactions are defined as any banking transactions (deposits or winnings) involving gambling. Deposits are defined as money transactions to gambling companies; winnings as transactions from gambling companies. The gambling balance/losses refer to deposits minus returned winnings.

We did not set a threshold for gambling consumption that would be considered excessive, but separately focused on the full sample and those with the highest gambling consumption in the past year. The highest gamblers were identified as the highest one and 5% of the total gambling consumption, with consumption defined as the sum of deposits or losses.

### Research ethics

Ethics approval for this research was obtained from the University of Helsinki Ethical Review Board in Humanities and Social and Behavioural Sciences (Statement 65/2022). Due to the sensitive nature of the data consisting of possibly identifiable bank transactions, the data were stored, and all analyses were conducted in a safeguarded network environment requiring a two-factor authentication. Only data relevant to the research question on the gambling of indebted individuals were transferred from Solidate to this research. This restricted the possibilities of research methodologies but was necessary to protect the personal data of Solidate customers who had not separately given consent to the research use of their data.

The analysis was not preregistered, and the results should be considered exploratory.

## Results

### Sample characteristics

Table 1 describes the past year gambling participation and loans of the individuals in the data. In total, 55.8% of the sample had gambled in the past year. Of those who have gambled, 94.6% have had a negative gambling balance in the last year, meaning they had lost more money than they had won. Furthermore, the median positive past-year balance is inferior in average monetary terms (median 790 euros, SD 35 125 euros) than the median negative past-year balance (median 1643 euros, SD 31 316 euros). The median past-year balance of individuals was negative by 1476 euros.

**Table 1:** Sample characteristics

	*N*	Percent
Gambling participation (full sample)		
Has gambled in the past year	12 963	55.8
No past year gambling	10 268	44.2
Total	23 231	100.0
Age (gambling sample)		
18–25	2355	18.2
26–35	3469	26.8
36–45	2111	16.3
46–55	1020	7.9
56–65	474	3.7
66–75	179	1.4
76–85	13	0.1
NA	3342	25.8
Total	12 963	100.0
Past year gambling balance (gambling sample)		
Positive	704	5.4
Negative	12 259	94.6
Total	12 963	100.0
Unsecured loans (gambling sample)		
Has unsecured loans	6503	50.2
No unsecured loans	6460	49.8
Total	12 963	100.0

The age distribution of individuals who had gambled is skewed towards younger age groups. Almost half of the total sample of past-year gamblers (45%) was under 35 years old, with a median age of 32. Half of the total sample of past-year gamblers (50.2%) also have unsecured debt.

As for transactions, gambling deposits make up over 90% of all gambling-related transactions in terms of numbers (*N*) and over 70% of gambling-related transactions in terms of money traffic. Paid winnings are fewer, but on average larger than deposits (median 50 euros, SD 3623 euros). Deposits are more frequent and smaller (median 20 euros, SD 166 euros).

### Concentration of spending

Past-year gambling spending is highly concentrated. Table 2 describes the past year gambling in the sample based on bank transactions. On average (median) individuals have deposited money on gambling accounts 62 times and the most frequently gambling 99th percentile have deposited on 2006 different occasions. In monetary terms, the median annual sum deposited is 2009 euros in the whole sample of past-year gamblers but 138 351 euros for the 99th percentile. If winnings are included, the top 5% (*N* = 650) have lost on average 77 422 euros in the past year, while the top 1% (*N* = 130) have lost on average 172 668 euros. The top 5% has spent over half of all the gambling money in the dataset (49.5% of deposits; 53.9% of losses).

**Table 2:** Past year gambling deposits of indebted individuals by percentiles

Gambler percentile	*N*	Sum (€)
25th	22	654
50th	62	2009
75th	262	8257
95th	1043	52 998
99th	2006	138 351
Mean	235	11 667
SD	421	40 228

### Gambling spending by product types

Part of the gambling deposits (33.2%) can be traced back to the type of gambling. Deposits and winnings by product category are described in table 3. Uncategorized deposits and winnings have been paid using online payment services. These are likely to be directed mostly online casino and sports betting websites. In terms of transactions that can be traced to a product category, casino-type gambling is the most popular among indebted individuals both in absolute terms and in terms of average payments. Casino-type gambling makes up 70% of categorized gambling spending, with sports betting making up 30% and lotteries <1%.

**Table 3:** Past year gambling spending of indebted individuals by product categories

	Sports betting	Casino	Lottery	Uncategorized
Deposits				
Euros (€)	15 087 393	34 820 024	36 296	100 441 165
*N*	643 812	655 230	1548	1 475 504
Median (€)	12	25	11	30
SD (€)	57.97	116.76	44.71	210.35
Winnings				
Euros (€)	10 851 904	22 667 640	32 358	23 300 582
*N*	114 094	79 434	552	78 445
Median (€)	20	100	15	100
SD (€)	5464.99	1221.50	183.16	838.39

A median deposit to a casino gambling service is 25 euros, in comparison to 12 euros for sports betting and 11 euros for lotteries. Casino-type gambling has also provided the largest on-average (median) winnings (100 euros), but these are much less frequent than outgoing payments. However, the return rate for sports betting appears to be higher (72%) than that of casino-type gambling (65%). The small number of transactions categorized under lottery complicate their comparison to the other formats.

### Gambling consumption and unsecured debt

The amount of unsecured debt is highly concentrated. The median amount of unsecured debt in the sample is 14 500 euros, but with wide variation (SD 25 456.72). The lowest quartile has an average of 4040 euros in unsecured debt, and the highest quartile has an average of 31 000 euros in unsecured debt. Table 4 compares gamblers in the sample with or without unsecured debt. Individuals with unsecured debt lose more money in gambling than those without in terms of sum figures of all past year gambling transactions (deposits and winnings). On average (median), individuals with unsecured debt have lost 1822 euros in gambling (SD: 23 809.97), while individuals without unsecured debt have lost 1204 euros in gambling (SD: 37 941.13). However, variations are important. Those with unsecured loans also have more gambling transactions. Individuals with unsecured debt have, on average (median), had 67 gambling transactions in the past year, compared to 56 in the group without unsecured debt.

**Table 4:** Past year gambling losses among individuals with and without unsecured loans

	Unsecured loans	No unsecured loans
Number of individuals	6503	6460
Past year gambling losses (€)	55 675 407	37 567 408
Past year gambling transactions (*N*)	1 691 256	1 357 869
Median losses (€)	1822	1204
Median transactions (*N*)	67	56
SD (€)	23 809.97	37 941.13

## Discussion

The current study has investigated the gambling of indebted individuals seeking debt consolidation services in Finland. Most previous research on gambling-related debt has used self-reported data. The current study has used objective banking transaction data. The results have shown that gambling is widespread among indebted individuals. In total, 55.8% of the sample have gambling-related banking transactions in the previous year. The rate is somewhat lower than the prevalence in the Finnish population study (78.2%). This is likely because gambling can result in a failed loan consolidation process, but also because of the absence of cash-based gambling in the dataset. Land-based electronic gambling machines (EGMs), lotteries, and scratch tickets are popular among Finnish gamblers. Thirty percent of past-year gamblers have gambled land-based only.^15^ EGMs can be paid for in cash, while scratch tickets can be bought in supermarkets as part of other shopping and cannot therefore be categorized as gambling in banking data.

In our sample, gambling spending is also highly concentrated. The highest spending 5% have spent half of all gambling in the sample. Gambling spending is more concentrated on individuals with unsecured loans in comparison those without unsecured loans. In terms of gambling types, casino-type gambling appears to be the most popular among indebted individuals. Based on the Finnish population study, casino-type gambling is also popular among those who experience gambling problems. Of those with a SOGS score of 3 or more, 14.4% have gambled on online EGMs and 19.4 have gambled at the casino in the past year.^15^

The results have important implications on financial harms of gambling. First, gambling is a significant contributor to indebtedness. While the correlation between gambling expenditure and debt does not necessarily equate to causation, the high gambling spending in the sample suggests that indebted individuals are vulnerable to gambling harms and that gambling is the likely reason for significant parts of individual (unsecured) debt. Accumulated debt may also encourage further gambling as a perceived solution. Qualitative accounts from gamblers suggest that debt is an outcome of excessive spending on gambling, but also a perceived motivator for continued gambling.^3^ However, most research links the accumulation of debt as well as other financial harms, directly to gambling.^18–20^

Second, the high levels of gambling behaviour particularly among those with unsecured loans raises concern regarding the intersection between harmful gambling and access to consumer credit. The wide availability of credit, and particularly instant loans, may be connected to excessive gambling spending and debt.^9^ Financial debt may partly stem from of poor money management or uncontrolled spending patterns,^4^ but these vulnerabilities are exploited by commercial interests both in the gambling and in the credit industries.^21^ The commercial determinants of harm involved in gambling span beyond the gambling industry and involve actors within the wider ecosystem of commercial interests related to excessive gambling.

Third, debt consolidation services appear to be at an important frontline position in terms of encountering gambling-related harms. Gambling problems are usually addressed by professional mental health support or by self-help groups. Yet, services providing information and help with financial issues appear to routinely encounter individuals that have a likely gambling problem and could benefit from brief screening tools for gambling.^22^ This has many practical implications. Partnership across different service providers is necessary to treat and prevent these harms. The large number of gambling-related transactions processed by online payment providers and financial service companies could also be used to identify problematic behavioural patterns (also^14^), similarly to efforts made with gambling company data in some contexts.

More research is needed on the processes and causal links that connect gambling and debt or other financial harms. Financial difficulties and debt may cause or aggravate other harms that have been connected to gambling, such as health issues, psychological suffering, family disfunction, or even suicide.^5^^,^^6^^,^^10^ Financial issues may therefore also function as mediators or catalysts for other harms. Problem gambling screening tools may not be optimal in the public health domain due to their focus on individual behavioural patterns rather than more systemic issues.^23^^,^^24^ More research is also needed on protective factors against debt-driven gambling, such as the role of financial literacy and money management.

The current study has been limited to a sample of indebted individuals in the context of Finland. The sample does not represent the full population of indebted individuals. Gambling is one of the leading causes for declined loan consolidation requests, making it likely that despite widespread gambling in the sample, this is an underrepresentation. Many individuals with excessive gambling are unlikely to even attempt contacting a service. As also observed in the UK, clients wait a long time before accessing debt services, and unwillingness to stop gambling is a major barrier for seeking help.^14^ The amounts of gambling in the sample may also be underestimated in the sample, as the banking transaction data cannot account for payments made in cash. In addition, not all payment service transactions could be classified. Debt-related services have access to unique data that could be further utilized for research, but this requires efforts in classifying and sharing data.^13^ Furthermore, the study has been limited by important restrictions to accessing variables regarding the socio-demographics of applicants. While this was necessary for reasons related to the protection of personal data, the lack of variables has placed limitations on the methods of analysis and interpretation and prevented investigating the role of many confounding factors, including family structures.

Nevertheless, bank transactions are an important source of data to assess individual gambling behaviour. Such data should be explored also in further studies as a possible alternative to, for example, data provided by gambling companies. In addition to cashless gambling cards (or personal gambling licences^25^) bank and payment service data can be used to improve the prevention of over-indebtedness as well as harmful gambling or related instant loan provision. Advances in digital technology have made access to credit easily available and debt fast to accumulate. This has the potential to further aggravate debt-related harms. Population-level, preventive measures are paramount in improving the health of populations and control policies to prevent these harms, including debt and financial distress.

## Data Availability

The data that support the findings of this study are available from the Finnish loan consolidation service Solidate, but restrictions apply to the availability of these data. The data were used under licence for the current study, and so are not publicly available. Data are however available from the authors upon reasonable request and with permission of Solidate.
